# Digestion

**Published:** 1879-10

**Authors:** 


### DIGESTION

Is that process which extracts from our food the elements of growth, repair, and sustenance. If the digestion is imperfect, the health of the body becomes imperfect in a few hours; and if by any means digestion ceases altogether, soon after a hearty meal, a man will as certainly die within a few hours, and sometimes almost as suddenly, as if a bullet were shot through his heart. Any great emotion of passion or pleasure, soon after eating, causes death; hence, no highly exciting or momentous news should be communicated, even to the healthiest, let alone the sick and the feeble, immediately after a full repast.

Sometimes the wisest of us will eat too much; for an occasional indiscretion of this kind, two or three teaspoonfuls of strong vinegar afford relief to some persons, but aggravate the evil in a few. The better plan is, to take a long leisure walk in the open air, with a pleasant associate. Keep on walking until entire relief is experienced, and eat no more of anything until next morning, so as to allow the overtaxed stomach to recover its tone, vigor, and elasticity.

If we become conscious of a surfeit after night, and from that or any other cause, a walk is impracticable, a good substitute is found, in standing erect with the clothing removed, except the stockings, mouth closed, and rubbing the region of the stomach, and for a foot around it, with the open hand. Very great relief is often afforded, even in serious cases, within half an hour, by a vigorous manipulation of this sort, taking for breakfast, next morning, a cup of some kind of hot drink and a single piece of dry bread; and for dinner, a bowl of soup with bread crust, and nothing else for that day. The stomach should always be allowed extra rest after overwork.

